# Enumeration of Functional T-Cell Subsets by Fluorescence-Immunospot Defines Signatures of Pathogen Burden in Tuberculosis

**DOI:** 10.1371/journal.pone.0015619

**Published:** 2010-12-14

**Authors:** Rosalyn Casey, Deena Blumenkrantz, Kerry Millington, Damien Montamat-Sicotte, Onn Min Kon, Melissa Wickremasinghe, Samuel Bremang, Murphy Magtoto, Saranya Sridhar, David Connell, Ajit Lalvani

**Affiliations:** 1 Tuberculosis Research Unit, Department of Respiratory Medicine, National Heart and Lung Institute, Imperial College London, London, United Kingdom; 2 Imperial College Healthcare NHS Trust, St Mary's Hospital, London, United Kingdom; Queensland Institute of Medical Research, Australia

## Abstract

**Background:**

IFN-γ and IL-2 cytokine-profiles define three functional T-cell subsets which may correlate with pathogen load in chronic intracellular infections. We therefore investigated the feasibility of the immunospot platform to rapidly enumerate T-cell subsets by single-cell IFN-γ/IL-2 cytokine-profiling and establish whether immunospot-based T-cell signatures distinguish different clinical stages of human tuberculosis infection.

**Methods:**

We used fluorophore-labelled anti-IFN-γ and anti-IL-2 antibodies with digital overlay of spatially-mapped colour-filtered images to enumerate dual and single cytokine-secreting *M. tuberculosis* antigen-specific T-cells in tuberculosis patients and in latent tuberculosis infection (LTBI). We validated results against established measures of cytokine-secreting T-cells.

**Results:**

Fluorescence-immunospot correlated closely with single-cytokine enzyme-linked-immunospot for IFN-γ-secreting T-cells and IL-2-secreting T-cells and flow-cytometry-based detection of dual IFN-γ/IL-2-secreting T-cells. The untreated tuberculosis signature was dominated by IFN-γ-only-secreting T-cells which shifted consistently in longitudinally-followed patients during treatment to a signature dominated by dual IFN-γ/IL-2-secreting T-cells in treated patients. The LTBI signature differed from active tuberculosis, with higher proportions of IL-2-only and IFN-γ/IL-2-secreting T-cells and lower proportions of IFN-γ-only-secreting T-cells.

**Conclusions:**

Fluorescence-immunospot is a quantitative, accurate measure of functional T-cell subsets; identification of cytokine-signatures of pathogen burden, distinct clinical stages of *M. tuberculosis* infection and long-term immune containment suggests application for treatment monitoring and vaccine evaluation.

## Introduction

Although T cell interferon-gamma release-assays (IGRA) are a major advance in diagnosis of LTBI, reliance on IFN-γ as the sole read-out means they provide only limited biological information which is clinically interpreted in a binary fashion, indicating either presence or absence of infection. A major limitation of current T cell-based diagnosis is the inability to monitor treatment response or distinguish active from latent TB [1]–[3].

Simultaneous measurement of IFN-γ and IL-2 at the single cell level identifies T cell cytokine profiles which reflect their memory phenotype and defines three main functional T cell subsets: effector cells that mainly secrete IFN-γ only, effector-memory cells primarily secreting both IFN-γ and IL-2, and central memory cells secreting only IL-2 [4]. These T cell subsets correlate with antigen and pathogen load *in vivo*, as exemplified by studies of chronic viral infections and tuberculosis (TB) and quantification of these key T cell subsets is a promising approach for diagnosis and monitoring of infectious diseases [5]–[10], as well as for identifying correlates of protective immunity [4], [11]–[19] and measuring vaccine immunogenicity [20]–[22].

The existing regulatory-approved commercial IGRAs are robust, extensively validated platforms for measuring the cell-mediated immune response to MTB antigens in routine clinical practice. However, measurement of IFN-γ as the sole marker of cellular immunity precludes exploiting the additional biological and clinical insights that could be provided by direct quantification of functional T cell subsets from patient blood samples. Given that the immunospot platform quantifies individual antigen-specific cytokine-secreting T cells, it might have potential for multiple cytokine profiling at the single cell level but development of dual-colour ELISpot has been hampered by the ambiguity of enumerating dual-colour spots due to masking of weaker signal from one colour-substrate by stronger signal from the second colour-substrate [23], [24]. Replacing enzymatic colorimetric signal with fluorophores emitting light at different wavelengths which are read through separate filters could in principle circumvent this problem. When each fluorophore-labelled monoclonal antibody detects a different cytokine [25], digital overlay of spatially-mapped images from the corresponding filters could enable detection of dual cytokine-secreting T cells based on co-localisation of different fluorophores [26] thereby generating a third, combined colour, or ‘footprint’ of dual cytokine-secreting cells.

We comprehensively examined for the first time the feasibility and accuracy of the membrane-based immunospot platform for rapid enumeration of the three key functional T cell subsets based on single-cell cytokine-profiling for IFN-γ and IL-2. We first validated this approach against established colour-ELISpot and flow-cytometry-based measures of single and dual cytokine secretion, respectively. We then hypothesised that fluorescence-immunospot would be able to distinguish distinct cytokine profiles in *M. tuberculosis* (MTB) antigen-specific T cells in different clinical stages of human TB infection, each cytokine signature being associated with different levels of pathogen and antigen load.

## Methods

### Participants

Ethical approval was granted by the NHS National Research Ethics Service, St Mary's Research Ethics Committee (London, UK) reference 07/H0712/85. Consenting adult patients were prospectively recruited from St Mary's Hospital, Imperial College Healthcare NHS Trust, London. Written informed consent was given in all cases.

The cross-sectional group of untreated active TB comprised 32 patients (table 1) and the cross-sectional group of treated TB comprised 24 patients sampled 3 or more months after commencing treatment (table 1). 6 of the patients recruited before treatment were also sampled after 3 months and included in the cross-sectional treated TB cohort. The nested longitudinal cohort comprised 19 of the untreated patients sampled pre-treatment and again 2-3 months later (6 patients at 3 months and 13 patients at 2 months). The LTBI group comprised 26 untreated persons diagnosed on the basis of positive IGRA results with one or more documented risk factors for TB exposure in whom active TB had been clinically and radiographically excluded [27]; 13 were recently infected and 13 remotely infected (see table 1 footnotes). 23 healthy BCG-vaccinated laboratory staff were recruited as healthy controls.

**Table 1:** Demographic and clinical characteristics of study participants.

Partcipant characteristics	UntreatedActive TB†(n = 32)	TreatedActive TB‡(n = 24)	Latent TB infection(n = 26)
Median age in years (range)	33 (21–60)	35.5 (19–69)	32 (18–79)
Female gender (%)	15 (46.8)	10 (41.6)	11 (42.3)
Ethnic Origin (%) -Asian	17 (53.1)	12 (50)	3 (11.5)
-Black African	8 (25)	8 (33.3)	11 (42.3)
-Caucasian	6 (18.8)	2 (8.3)	7 (26.9)
-Other	1 (3.1)	2 (8.3)	5 (19.2)
Exposure to active TB case (%) - Recent*	n/a	n/a	13 (50)
- Remote**	n/a	n/a	13 (50)
BCG Vaccination status (%) -Vaccinated	25 (78.1)	20 (83.3)	15 (57.7)
-Unvaccinated	5 (15.6)	3 (12.5)	8 (30.8)
-Unknown	2 (6.3)	1 (4.2)	3 (11.5) (0)
Clinical characteristics at time of diagnosis			
MTB culture positive where tested (%)	18/30 (60)	11/22 (50)	n/a
Histological evidence of granulomas where tested (%)	12/18 (66.7)	10/17 (58.8)	n/a
Radiological evidence of TB where tested (%)	26/30 (86.7)	21/23 (91.3)	n/a
Site of disease (%) - Lymphatic	15 (46.9)	11 (45.8)	n/a
-Pulmonary	11 (34.4)	7 (29.2)	n/a
-Pleural	1 (3.1)	2 (8.3)	n/a
-Disseminated	4 (12.5)	3 (12.5)	n/a
-Extrapulmonary	1 (4.2)	1 (4.2)	n/a
TST positive where tested (%)	23/27 (85.2)	18/21 (85.7)	17/21
IGRA positive where tested (%)	15/15 (100)	11/11 (100)	26/26 (100)
Median length of treatment in months (range)	0 (0–0.3)	6 (3–9)	0

† 5 patients with 1–4 days treatment and 2 with 1–2 weeks treatment at recruitment.

‡ 11 patients at 3 months, 9 at 4–6 months and 7 at 7–9 months after initiating treatment.

*Recent infection: persons exposed to an infectious TB case less than 6 months before recruitment and resident in the UK for more than 2 years.

**Remote infection: persons who immigrated to the UK from a high-incidence country more than 2 years ago with no known exposure to TB within the last 2 years.

nt  =  not tested.

### Purification of lymphocytes from peripheral blood

PBMC were isolated from 40 millilitres of heparinised whole blood less than 4 hours after drawing by Ficoll-Paque^PLUS^ density centrifugation (GE Healthcare, Amersham, UK) washed twice in RPMI 1640 (Sigma-Aldrich, Irvine, UK), and cryopreserved in heat-inactivated foetal calf serum supplemented with 10% DMSO (Sigma-Aldrich). After no more than 12 months storage in liquid N_2_, cells were thawed at 37°C for one minute, washed twice in RPMI supplemented with 10% foetal calf serum and resuspended in AIM-V media (Invitrogen, Paisley, UK) for viability checking by trypan blue exclusion. Sample viability ranged from 80–98% and cells were used in assays with no rest period.

### Ex vivo colour IFN-γ ELISpot

Pre-coated plates (Mabtech, Nacka, Sweden) were equilibrated with AIM-V media for 30 min before seeding. Duplicate wells were seeded with 2.5×10^5^ PBMCs in AIM-V and contained: no peptide, 5 µg/ml PHA (MP Biomedicals, Illkirch, France), 20 µg/ml tuberculin PPD (Satens Serum Institute, Copenhagen, Denmark) or peptide pools containing 15-mer peptides overlapping by 10 amino acids (10 µg/ml final concentration; Pepceuticals Ltd, Nottingham, UK) spanning the length of ESAT-6 (17 peptides) or CFP-10 (18 peptides). Plates were incubated for 18 hours at 37°C with 5% CO_2_ then washed 5 times with 200 µl/well PBS and developed for one hour with 100 µl/well ALP-conjugated IFN-γ detection antibody diluted to manufacturer's instructions (Mabtech) before washing 5 times with PBS and incubating for 7 minutes with 50 µl/well BCIP/NBT^PLUS^ (Moss, Pasadena, MD, USA) and thoroughly washed with water, as previously described [(ref28)].

Spot-forming cells (SFCs) were counted using automated ELISpot reader (Autoimmun Diagnostika GmbH, Strassberg, Germany) with pre-defined threshold size and intensity settings (see Table S1).

### Ex vivo colour IL-2 ELISpot

The ex-vivo IL-2 ELISpot was performed in the same manner as the ex-vivo IFN-γ-ELISpot with the following differences, Plates (Multiscreen_HTS_^TM^ IP, Millipore, Billerica, MA) were activated with 15 ul/well 35% ETOH for one minute before washing 5 times with 200 µl/well sterile water and coating overnight at 4°C with 10 ug/ml coating IL-2 antibody (Mabtech). Plates were washed 5 times with 200 µl/well sterile PBS before seeding with cells and antigen as per IFN-γ-ELISpot assay. After 18 hours incubation, plates were washed and developed for 2 hours with 100 µl/well of 2.5 µg/ml biotinylated anti-IL-2 antibody (IL2-II-biotin, Mabtech) followed by five PBS washes and 100 µl/well of streptavidin-ALP diluted to manufacturer's instructions (Mabtech) for one hour. After 5 PBS washes, plates were incubated for 20 minutes with 50 µl/well BCIP/NBT^PLUS^ and washed thoroughly with water. SFCs were counted using the same ELISpot reader as for IFN-γ-ELISpot with separate pre-defined threshold size and intensity settings (see Table S1).

### Ex vivo IFN-γ/IL-2 fluorescence-immuospot

Plates (Multiscreen_HTS_TM IPFL, Millipore, Billerica, MA) were activated with 15 ul/well 35% ETOH for one minute before washing 5 times with 200 µl/well sterile water and coating overnight at 4°C with 20 µg/ml anti-IL-2 and 20 µg/ml anti-IFN-γ antibodies (Mabtech). Duplicate wells were seeded with PBMCs and incubated with test and control conditions exactly as for colour ELISpot but with the addition of anti-CD28 IgG antibody (Mabtech, final concentration 0.1 µg/ml). After 18 hours, plates were washed then incubated with 2 µg/ml monoclonal FITC-conjugated anti-IFN-γ (7-B6-FITC, Mabtech) and 2.5 µg/ml biotinylated anti-IL-2 detection (IL2-II-biotin, Mabtech) antibodies. After washing, plates were incubated with fluorophore-labelled secondary antibodies, anti-FITC-PF488P and streptavidin-PF555 (Mabtech) at 1 µg/ml, washed again and incubated at room temperature for 15 minutes with 100 µl/well fluorescence enhancer (Mabtech) before being emptied and air-dried.

SFCs were counted by automated fluorescence plate reader (iSpot, Autoimmun Diagnostika GmbH) fitted with colour filters for FITC and Cy3 which detect PF488P and PF555 fluorophores respectively. iSpot software version 5 was used to count plates using pre-defined threshold size and intensity settings for spots (see Table S1 for full instrument settings). The iSpot software digitally overlays single stained images to give a third dual-stained image for the enumeration of dual-cytokine-secreting cells. The rate of false-positive reporting of dual-spots (due to co-localization of spots from different cells) by the iSpot automated plate reader is <2% when single-stained spots per well are 500 or less, which was the case for 98.5% of responses in our dataset (Autoimmun Diagnostika, unpulblished data on file).

### Positive response threshold settings for immunospot assays

**Figure 1:**
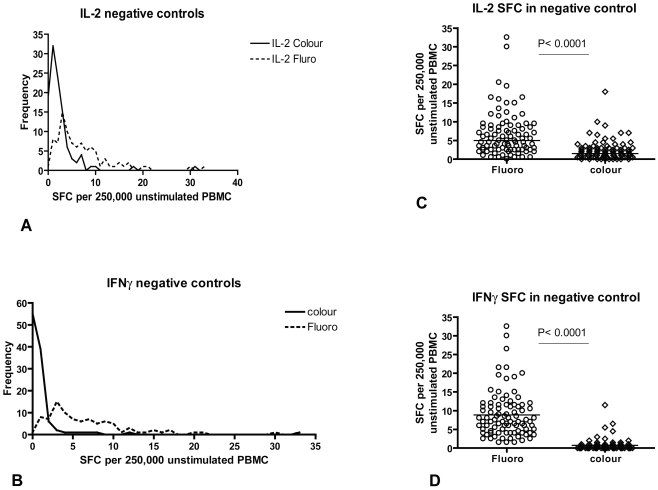
Comparison of number of spot-forming-cells (SFC) found in negative control wells of fluorescence-immunospot and colour ELISpot. Overlaid histograms of number of spot-forming-cells (SFC) found in negative control wells of colour ELISpot and fluorescence-immunospot measuring IL-2 (A) and IFN-γ (B). Median values for IL-2 SFC and IFN-γ SFC were higher in the fluorescence-immunospot compared to the colour ELISpot (C & D, both P<0.0001). This was attributed to the use of co-stimulatory anti-CD28 antibody in the fluorescence-immunospot to overcome the effects of sequestering of IL-2 by membrane-bound IL-2 antibodies. The resultant increased background cytokine-secretion in negative control wells was adjusted for by subtracting negative control responses from antigen-specific responses.

Antigen-specific responses were calculated by subtracting the average number of SFCs in duplicate donor-specific negative control wells from SFCs in test wells for each donor. Thresholds for a positive response for each cytokine were pre-defined as two standard deviations above the mean of all negative control wells (figure 1). This equated to 8 IFN-γ SFCs and 12 IL-2 SFCs more than the negative control wells for each patient for fluorescence-immunospot and 5 IFN-γ SFCs and 8 IL-2 SFCs more than the negative control wells for colour ELISpot. This threshold was chosen in order to encompass 95% of variation within negative controls (figure 1). Positive ESAT-6 and CFP10 responses were summated for each participant to give ESAT-6/CFP-10 responses.

### Cytokine secretion assay

PBMCs from healthy BCG-vaccinated donors were incubated in the presence of no antigen, PPD (20 µg/ml) or Staphylococcal Enterotoxin B (SEB, Sigma, 4 ng/ml data not shown) for 4 hours at 37°C (3 million cells per condition). PBMCs were then washed twice with MACS buffer (PBS, 0.5% BSA and 2 mM EDTA (Sigma-Aldrich)) and centrifuged for 10 minutes at 4°C. Cell pellets were resuspended in 80 µl per test condition of cold RH5 (RPMI supplemented with 5% heat-inactivated human AB serum (Sigma-Aldrich)). Ten microlitres of IFN-γ catch reagent and 10 µl of IL-2 catch reagent (Miltenyi Biotec, Bergisch Gladbach, Germany) were added per test condition and incubated on ice for 5 minutes. Five millilitres of warm RH5 was added and cells rotated at 37°C in 5% CO_2_ for 45 min; cells were then incubated on ice for 10 min in 9 ml ice-cold MACS buffer before centrifugation and resuspension in 80 µl cold MACS buffer. After staining with 10 µl allophycocyanin-conjugated IL-2-specific detection antibody, 10 µl FITC-conjugated IFN-γ-specific detection antibody (Miltenyi Biotec) and 3 µl phycoerythrin-conjugated anti-CD3 antibody (BD Biosciences, Oxford, UK) for 15 min on ice. Cells were washed twice and resuspended in 100 µl MACS buffer. Dead cells were excluded by staining with 7-amino-actinomycin-D (BD Biosciences) just before acquisition by flow-cytometry on an LSR II flow-cytometer (BD Biosciences) and analysed using FlowJo software (Tree Star, Ashland, OR). At least 300,000 events were captured per sample. The number of PPD-specific IL-2/IFN-γ secreting cells per million CD3^+^ lymphocytes was calculated by subtracting the number of dual-stained unstimulated CD3^+^ lymphocytes from the number of dual-stained PPD-stimulated CD3^+^ lymphocytes. To derive the number of cytokine secreting T cells per million PBMC, the number of PPD-specific CD3^+^ lymphocytes was multiplied by the proportion of PBMC that were CD3^+^ for each donor.

### Statistical analysis

Spearman's rank correlation was used to measure correlation between data from fluorescence-immunospot and pre-existing assays with 99% confidence intervals. Mann-Whitney 2-tailed U test was used to identify statistically significant differences between groups. GraphPad prism version 4 was used for statistical analysis. The range of absolute frequencies of the antigen-specific functional T cell subsets across individuals and inter-individual variation were considerable and frequencies of IFN-γ-only, IL-2-only and dual cytokine-secreting T cells in each donor were therefore analysed as proportions.

Data can be made available to academic investigators on written request by agreement with Prof A. Lalvani at a.lalvani@imperial.ac.uk.

## Results

### Demographic and clinical characteristics of study participants

Demographic and clinical characteristics of the study participants are summarized in table 1. Patients were categorised into three clinical groups: untreated active TB (n = 32), treated active TB (n = 24) and untreated latent TB infection (n = 26). There were no significant differences in age, gender, ethnicity or BCG vaccination status between the groups, except that the latently infected group contained a lower proportion of Asians. No LTBI donors had subclinical active TB as none developed active disease during 12 months follow-up. Our study population has a very low (4%) prevalence of HIV-coinfection and no participants had any clinical or laboratory features suggestive of HIV infection [29].

### Enumeration of IFN-γ and IL-2 -secreting cells by fluorescence-immunospot correlates closely with measurement by colour ELISpot

PBMC from 90 independent blood samples from 76 subjects with active TB or latent TB infection were incubated with media alone or stimulated with peptides from the MTB-specific proteins early secreted antigenic target 6 (ESAT-6), culture filtrate protein-10 (CFP-10) or purified protein derivative (PPD). Secretion of the two cytokines was measured in parallel by colour IFN-γ-ELISpot, colour IL-2-ELISpot and fluorescence-immunospot. Numbers of SFCs in negative control wells in the parallel assays are compared in figure 1. The fluorescence-immunospot negative control wells had significantly higher numbers of IL-2 (A and C) and IFN-γ positive spots (B and D) compared to the colour ELISpots. The mean number of negative control SFC/well was used to calculate assay-specific thresholds for positive responses (see Methods). PBMC from 8 BCG-vaccinated healthy controls were also stimulated with the same panel of antigens in the fluorescence-immunospot assay and no positive responses to ESAT-6 or CFP-10 were detected (data not shown).

**Figure 2:**
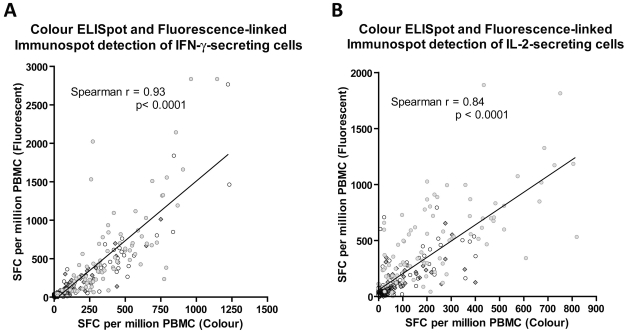
Comparison of the number of antigen-specific spot-forming cells (SFC) detected by fluorescence-immunospot and colour ELISpot. PBMC from 90 independent samples from 76 donors were stimulated with PPD (diamonds), ESAT-6 (hollow circles) or CFP-10 (grey filled circles). IFN-γ (A) or IL-2 producing cells (B) were enumerated by colour ELISpot and fluorescence-immunospot. Correlation between results of the 2 tests was tested by Spearman's rank correlation. Responses to PPD and peptide pools of ESAT-6 and CFP-10 were each strongly correlated between the colour and fluorescent assays for enumerating IFN-γ-secreting cells (r = 0.89 CI: 0.83–0.94, r = 0.89 CI: 0.9–0.95, r = 0.83 CI: 0.72–0.90 for PPD, ESAT-6 and CFP-10 respectively) and IL-2-secreting cells (r = 0.84 CI: 0.73–0.90, r = 0.73 CI: 0.56–0.84, r = 0.70 CI: 0.53–0.82 for PPD, ESAT-6 and CFP-10 respectively).

Numbers of IFN-γ and IL-2 SFCs enumerated by the colour and fluorescent assays were strongly and significantly correlated (Figure 2, Spearman's rank r = 0.93 and 0.84 respectively, p<0.0001 for both cytokines). The slope of the linear relationship was 1.55 for IFN-γ and 1.45 for IL-2; the higher numbers of IFN-γ and IL-2 SFCs detected in the fluorescence-immunospot compared to conventional colour ELISpot are attributable to co-stimulation of antigen-specific T cells resulting from inclusion of co-stimulatory anti-CD28 IgG antibody in our protocol (see Methods) to counterbalance sequestering of IL-2 by membrane-bound anti-IL-2 capture antibody [30]. The resultant increased background cytokine-secretion in negative control wells was adjusted for by subtracting negative control responses from antigen-specific responses.

For both IFN-γ-secreting and IL-2-secreting T cells, responses to PPD and ESAT-6 and CFP-10 peptide pools were equally strongly correlated between the colour and fluorescent immunospot assays (Figure 2) indicating that inter-assay correlation is independent of the antigen used.

Comparing positive and negative responses for all 3 antigens between the fluorescent and colour assays revealed that 90% of IFN-γ results and 87% of IL-2 results were concordant (Table S2).

### Enumeration of dual IFN-γ/IL-2 secreting T cells by fluorescence-immunospot gives equivalent values to measurement by direct cytokine staining and flow-cytometry

**Figure 3:**
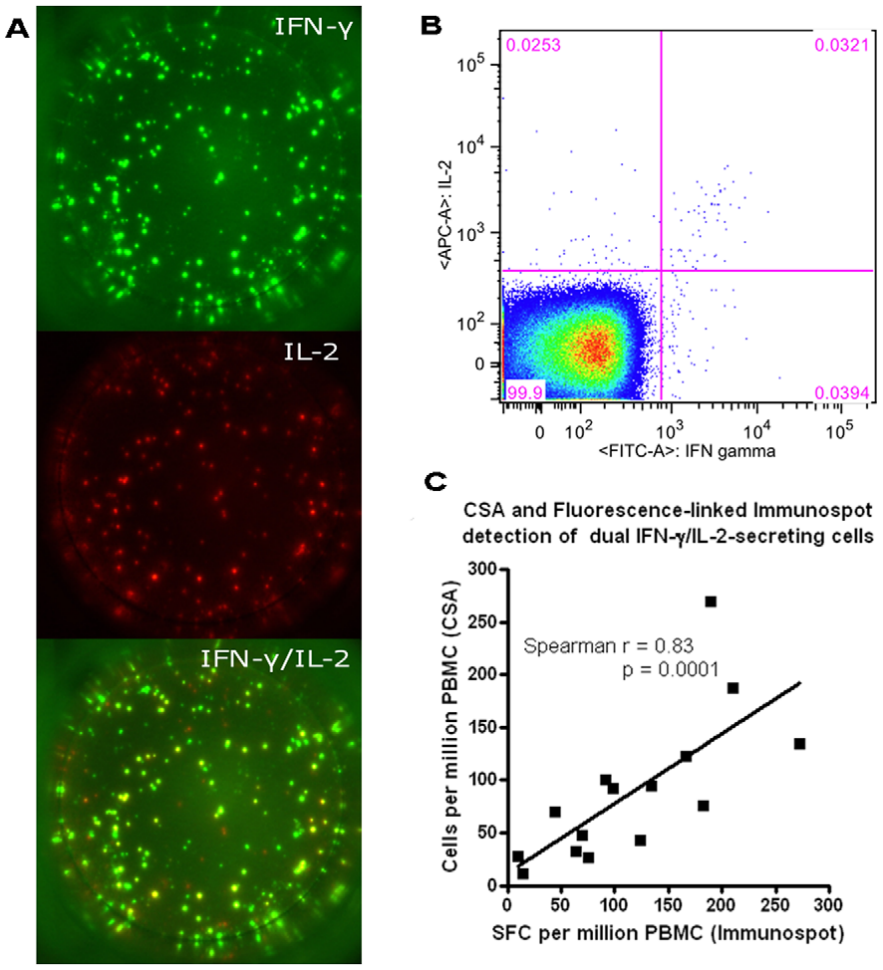
Comparison of the number of dual IFN-γ/IL-2 –secreting cells detected by cytokine secretion assay (CSA) and fluorescence-immunospot. PBMC from 15 healthy BCG-vaccinated volunteers were stimulated with PPD and IFN-γ–secreting, Il-2-secreting and dual IFN-γ/IL-2–secreting cells were enumerated by CSA (gating on CD3^+^ lymphocytes) and fluorescence-immunospot. Fluorescence-immunospot IL-2, IFN-γ and digitally-overlaid dual images from a representative donor are shown in A with CSA results from the same donor shown with percentage of cells stated in each quadrant shown in B. Correlation between the numbers of dual IFN-γ/IL-2-secreting cells detected by the 2 tests was tested by Spearman's rank correlation (C).

To confirm that fluorescence-immunospot accurately measures dual-cytokine secreting cells, we compared the number of PPD-specific IFN-γ/IL-2-secreting cells enumerated by fluorescence-immunospot with numbers detected by dual staining using the cytokine secretion assay (CSA) in PBMC from 15 BCG-vaccinated healthy donors. Figure 3a shows fluorescence-immunospot IL-2, IFN-γ and digitally-overlaid dual images from a representative donor with CSA results from the same donor shown in figure 3b. The number of dual cytokine-secreting SFC detected by fluorescence-immunospot across the 15 donors was strongly and significantly correlated with the number of dual-cytokine secreting cells detected by CSA (Spearman's rank, r = 0.83, p = 0.0001) (figure 3c).

### Fluorescence-immunospot identifies distinct T cell cytokine signatures in untreated and treated TB patients

We used the validated fluorescence-immunospot to quantify and compare functional T cell subsets in TB patients before and during treatment. Median frequencies of total cytokine-secreting T cells specific for ESAT-6/CFP-10 peptides (248 (IQR 96-860) per million PBMC) and PPD (524 (IQR 196-1152)) in untreated TB patients were not significantly different from those in treated patients (268 (IQR 128-748) for ESAT-6/CFP-10 and 728 (IQR 324-1044) for PPD) (p = 0.76, p = 0.35, respectively).

**Figure 4:**
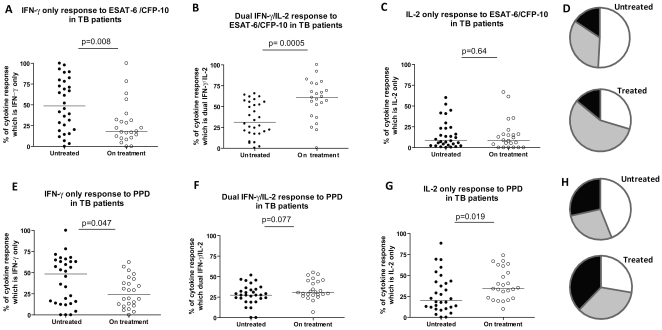
Cytokine responses to ESAT-6/CFP-10 and PPD in patients with Active TB before (n = 32) and during treatment (after 3–9 months of treatment, n = 24). PBMC from patients with active TB were stimulated with ESAT-6/CFP-10 (A-D) or PPD (E-H) and the number of IFN-γ-only secreting cells (A,E) dual IFN-γ/IL-2 –secreting cells (B,F) and IL-2-only secreting cells (C,G) were enumerated by fluorescence-immunospot. Data are expressed for all responding patients as the percentage of the total number of cytokine-secreting cells detected which were IFN-γ-only, IL-2-only or dual IFN-γ/IL-2 –secreting cells. Mean proportions of ESAT-6/CFP-10–specific and PPD-specific cytokine-secreting cells for each patient group are depicted as pie charts in figures D and H respectively (where white =  IFN-γ only, Grey  =  IFN-γ/IL-2 and black  =  IL-2 only-secreting cells).

The proportions of ESAT-6/CFP-10 and PPD-specific cytokine-secreting T cells that were IFN-γ-only, dual-IFN-γ/IL-2 or IL-2-only in the cross-sectional cohorts of untreated TB versus treated patients are shown in figure 4. TB patients on treatment had a significantly higher proportion of ESAT-6/CFP-10-specific IFN-γ/IL-2 secreting T cells and a significantly lower proportion of ESAT-6/CFP-10-specific IFN-γ-only secreting cells than the untreated cohort (figure 4a, b & d, p = 0.0005 and p = 0.008 respectively) with no significant difference in the proportion of ESAT-6/CFP-10-specific IL-2 only-secreting cells (figure 4c). The difference in the proportions of PPD-specific IFN-γ/IL-2-secreting T cells between untreated and treated patients was not statistically significant (figure 4f, p = 0.077); however, the proportion of PPD-specific IFN-γ-only-secreting T cells was significantly higher in untreated patients (figure 4e, p = 0.047) while the proportion of IL-2-only secreting T cells was significantly lower (figure 4g p = 0.019).

### Fluorescence-immunospot reveals consistent changes in T cell cytokine signatures within individual TB patients tracked longitudinally during treatment

**Figure 5:**
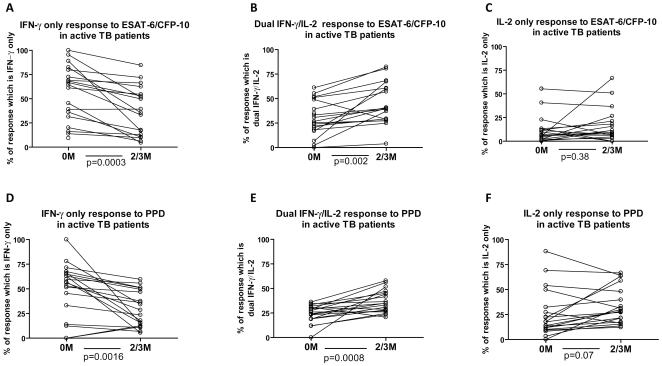
Cytokine responses to ESAT-6/CFP-10 and PPD in patients with active TB followed longitudinally before and during treatment (2 or 3 months of treatment). PBMC from 19 patients with active TB were isolated before treatment and after 2 or 3 months treatment and stimulated with ESAT-6/CFP-10 (A–C) or PPD (D–F) and the number of IFN-γ-only secreting cells (A,D) Dual IFN-γ/IL-2–secreting cells (B,E) and IL-2-only secreting cells (C,F) were enumerated by fluorescence-immunospot. Data are expressed for all responding patients as the percentage of the total number of cytokine-secreting cells detected which were IFN-γ-only, IL-2-only or dual IFN-γ/IL-2–secreting cells.

Nineteen untreated active TB patients were re-tested after 2–3 months treatment (figure 5): median frequencies of total cytokine-secreting T cells specific for ESAT-6/CFP-10 peptides and for PPD per million PBMC were 388 (IQR 148-972) and 792 (IQR 124-1276) pre-treatment and 132 (IQR 88-952) and 916 (IQR 324-1120) after 2-3 months treatment, respectively. The change in total cytokine-secreting T cells specific for ESAT-6/CFP-10 (p = 0.03), but not for PPD (p = 0.66), was statistically significant.

Eighteen of 19 patients displayed a decrease in the proportion of IFN-γ-only secreting ESAT-6/CFP-10-specific T cells after treatment (figure 5a, p = 0.0003). The proportion of ESAT-6/CFP-10-specific IFN-γ/IL-2-secreting cells increased significantly after treatment in all but one patient (figure 5b, p = 0.002). There was no significant change in the proportions of ESAT-6/CFP-10-specific IL-2-only-secreting cells which remained low (under 30%) in all but 3 patients (figure 5c). The proportion of PPD-specific IFN-γ-only-secreting cells decreased significantly during treatment and the proportion of dual IFN-γ/IL-2-secreting cells increased significantly (figure 5d-e, p = 0.0016 and p = 0.0008 respectively). The proportion of PPD-specific IL-2-only-secreting cells increased non-significantly in the majority of patients during treatment (figure 5f, p = 0.07). All 19 patients responded successfully to anti-tuberculous therapy and the consistent immunological changes observed were associated with consistent improvements in clinical status (weight gain and resolution of presenting symptoms and pyrexia where present), microbiologic status (conversion of sputum cultures to negative in the 12 patients who were initially culture-positive) and radiological findings on chest radiography and computerised axial tomography.

### Fluorescence-immunospot identifies distinct T cell cytokine signatures that distinguish LTBI from active TB

Median absolute frequencies of total cytokine-secreting T cells specific for ESAT-6/CFP-10 peptides and for PPD per million PBMC were 248 (IQR 96-860) and 524 (IQR 196-1152) in untreated active TB and 364 (IQR 128-1164) and 724 (IQR 380-1400) in the group with LTBI, respectively, with no significant difference between active TB patients and LTBI (p = 0.84, p = 0.14, respectively).

**Figure 6:**
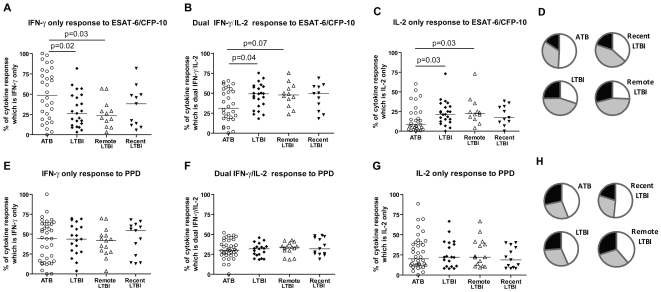
Cytokine responses to ESAT-6/CFP-10 and PPD in untreated active TB patients and persons with latent TB infection (LTBI). PBMC from 32 untreated patients with Active TB (ATB, circles) and 26 persons with LTBI (diamonds) were stimulated with ESAT-6/CFP-10 (A–D) or PPD (E–H) and the number of IFN-γ-only secreting cells (A,E) dual IFN-γ/IL-2 –secreting cells (B,F) and IL-2-only secreting cells (C,G) were enumerated by fluorescence-immunospot. LTBI data was also subdivided into persons with recent exposure (black triangles, n = 13) and those with remote exposure to an infectious TB case (hollow triangles, n = 13). Data are expressed for all responding patients as the percentage of the total number of cytokine-secreting cells detected which were IFN-γ-only, IL-2-only or dual IFN-γ/IL-2-secreting cells. P values are stated for comparisons of ATB with all LTBI and remote LTBI only. There was no statistically significant difference between the proportions of IFN-γ-only, IL-2-only or dual IFN-γ/IL-2 –secreting ESAT-6/CFP-10-specific cells in ATB and recent LTBI (p = 0.15, p = 0.16, p = 0.18, respectively). There was no statistically significant difference between the proportions of any PPD-specific cytokine-secreting cells (data not shown). Mean proportions of ESAT-6/CFP-10–specific and PPD-specific cytokine-secreting cells for each patient group are depicted as pie charts in figures D and H respectively (where white =  IFN-γ only, Grey  =  IFN-γ/IL-2 and black  =  IL-2 only-secreting cells).

Having determined that the MTB-specific T cell cytokine profile distinguishes the low *in vivo* antigen load state of treated TB from the high antigen load state pre-treatment, we postulated that it would also distinguish the low pathogen load state of LTBI from untreated active TB. Untreated active TB patients had a significantly higher proportion of ESAT-6/CFP-10 specific IFN-γ only-secreting T cells and a significantly lower proportion of ESAT-6/CFP-10 specific IFN-γ/IL-2-secreting and IL-2 only-secreting T cells than persons with untreated LTBI (figures 5a, b, c, d, p = 0.02, p = 0.04 and p = 0.03 respectively). After stratification for duration of latent infection, the differences from active TB in the single cytokine-secreting ESAT-6/CFP-10-specific functional T cell subsets remained significant for the remotely-infected (p = 0.03, p = 0.07 and p = 0.03 for IFN-γ-only-secreting, IFN-γ/IL-2-secreting and IL-2-only-secreting T cells, respectively) but not the recently-infected subgroup (p = 0.15, p = 0.16, p = 0.18, respectively). In contrast, functional T cell subsets specific for PPD were not significantly different between untreated active TB and LTBI (figure 6e, f, g, h). Functional T cell subsets specific for PPD or ESAT-6/CFP-10-specific were not significantly different between the two LTBI subgroups although this comparison was based on low numbers (n = 12 remote vs. n = 11 recent for ESAT-6/CFP-10 responders).

## Discussion

Our findings establish fluorescence-immunospot as a sensitive, high-throughput platform to enumerate antigen-specific functional T cell subsets directly *ex vivo* and validate this platform in the clinical setting for the first time. We verified that fluorescence-immunospot gives equivalent results to its colour-based ELISpot predecessor for detection of IFN-γ and IL-2 secretion by individual antigen-specific T cells, independently of the antigen used. Furthermore, absolute frequencies of dual cytokine-secreting T cells enumerated by fluorescence-immunospot were tightly correlated with those enumerated by the flow-cytometric-based method of CSA, confirming that digital co-localisation of red and green SFCs in fluorescence-immunospot is an accurate measure of dual cytokine-secreting T cells. Although strongly correlated with ELISpot, frequencies of antigen-specific T cells enumerated by fluorescence-immunospot were generally somewhat higher due to the use of anti-CD28 for costimulation, as is commonly done in ICS. However, negative control wells also included anti-CD28 and thresholds for positive results were derived from these wells; the impact of co-stimulation was thus taken into account by our methodology and concordance for positive and negative results between the assays was high.

Following validation, we used fluorescence-immunospot to identify signatures of functional T cell subsets corresponding to the differing pathogen and antigen loads in distinct clinical stages of TB infection. The proportion of ESAT-6/CFP-10-specific IFN-γ-only secreting cells was significantly lower in treated TB patients compared to untreated patients and decreased in 18 of 19 patients followed longitudinally during treatment. Conversely, the proportion of ESAT-6/CFP-10-specific IFN-γ/IL-2-secreting cells was significantly higher in treated compared to untreated TB patients and increased in 18 of 19 patients followed longitudinally.

Studies with IFN-γ release assays (IGRAs) have shown that ESAT-6 and CFP-10-specific IFN-γ-secreting T cells often decline at the population level in TB patients during treatment, but this trend masks substantial inter-individual variation with some patients showing no decline and others an increase [3], [6], [31]-[34] making IGRAs unsuitable for monitoring treatment response or test of cure [3], [6], [35]. However, dissecting out the composition of ESAT-6/CFP-10-specific IFN-γ-secreting T cell populations in patients during the substantial reductions in pathogen load induced by the induction phase of anti-TB treatment revealed remarkably consistent dynamic changes in the underlying IFN-γ-only-secreting T cells and dual IFN-γ/IL-2-secreting T cells that together comprise the overall IFN-γ response, with the former declining and the latter increasing in 18 out of 19 patients. Given that all 19 patients responded well to anti-TB treatment these dynamic MTB-specific T cell functional signatures suggest a biomarker of treatment response and decreasing mycobacterial load. Ours is the only study of functional T cell subsets to present consistent pair-wise data for individual patients over time. The only other study longitudinally to track MTB-specific functional T cell subsets in TB patients, by Caccamo et al, used flow-cytometry and also found a significant increase in dual IFN-γ/IL-2-secreting T cells after treatment for the study population as a whole [36]. Although the shift towards dual IFN-γ/IL-2-secreting T cells is emerging as a promising biomarker of treatment response, large-scale clinical studies are now required to determine its clinical utility and should include patients who fail to respond to treatment such as those with multidrug-resistant TB. Caccamo et al also measured TNF-α and found a reduction in tri-functional ESAT-6/CFP-10-specific T cells secreting IFN-γ, IL-2 and TNF-α with treatment and, in contrast to the present study and our earlier cross-sectional study [6], an increase in IFN-γ-only-secreting T cells.

IL-2–only secretion is indicative of long-lived central-memory T cells and there was no significant increase in these cells at 2–3 months treatment in contrast to the findings in patients at 6–28 months post-treatment using CSA with enrichment [6], suggesting that several months may be required for circulating MTB-specific IL-2-only secreting T cells to increase. In contrast, the proportion of PPD-specific IL-2 only-secreting T cells increased significantly in the treated compared to the untreated patients and increased non-significantly in the longitudinal cohort at 2–3 months, suggesting that the kinetics of emergence of PPD-specific central-memory cells during treatment are faster than ESAT-6/CFP-10-specific T cells. The reasons for this difference are unclear but might relate to the fact that PPD-specific IL-2-secreting T cells include cross-reactive memory T cells generated by prior BCG vaccination.

We hypothesised that, in contrast to active TB, latently infected individuals would display an immunological signature consistent with persistent low antigen load and long-term immune control, i.e. one dominated by dual IFN-γ/IL-2 secreting effector-memory T cells accompanied by IL-2-secreting central memory T cells. Compared to active TB patients, the LTBI donors had a significantly lower proportion of ESAT-6/CFP-10-specific IFN-γ-only secreting T cells and significantly higher proportions of ESAT-6/CFP-10-specific dual IFN-γ/IL-2-secreting and IL-2-only secreting T cells, confirming our hypothesis. Consistent with our results, two recent studies comparing MTB-specific functional T cell subsets in active TB and LTBI using ICS and flow-cytometry for IFN-γ, IL-2 and TNF-α similarly found that ESAT-6/CFP-10-specific IL-2-only secreting T cells were significantly increased in latently infected household contacts in The Gambia [37] and ESAT-6/CFP-10-specific dual IFN-γ/IL-2-secreting T cells were increased in latently infected healthcare workers in Italy [36]. Thus, MTB-specific T cells secreting IL-2 (with or without IFN-γ) are preferentially associated with LTBI compared to active TB, consistent with a role in long-term immune control. This interpretation is also supported by studies in LTBI with HIV co-infection, where advancing immunosuppression (and risk of active TB) are associated with declining MTB-specific IL-2 and dual IFN-γ/IL-2-secreting T cells [38], [39] while immune reconstitution is accompanied by increasing MTB-specific IL-2-secreting central-memory and dual IFN-γ/IL-2-secreting T cells but decreasing proportions of IFN-γ-only secreting T cells [9], [40], [41]. Notably, however, MTB-specific T cells simultaneously secreting IFN-γ, IL-2 and TNF-α are higher in active TB than LTBI which argues against a role for tri-functional cells as mediators of long-term immune control in humans [36].

Stratification of our LTBI cohort into subgroups that were recently or remotely infected with MTB suggested that the differences in T cell cytokine signatures between active and latent TB were driven by the remotely-infected group who have more of a central-memory T cell functional signature than persons recently infected with MTB. Ours is the first analysis of functional T cell subsets in epidemiologically distinct phenotypes of latent infection and now requires corroboration in a larger study population to ascertain whether significant differences in cytokine signature exist between recent and remote infections. Of note, enumeration of ESAT-6/CFP-10-specific IFN-γ-producing T cells by conventional IFN-γ ELISpot did not detect immunological differences between active TB, recent LTBI and remote LTBI other than an overall increase in IFN-γ-producing T cells in active TB [42].

The robustness and simplicity of the fluorescence-immunospot makes it a promising tool for treatment and vaccine trials in resource-poor settings. Although advanced flow-cytometry is increasingly available in laboratory research field stations in the developing world, the ELISpot platform is generally more widely available in these settings being less expensive and requiring much less equipment. We believe that save for the cost of upgrading to fluorescence-filters, fluorescence-immunospot is as suitable to infectious disease and immunological investigations in field sites in resource-poor settings as ELISpot.

This study establishes fluorescence-immunospot as a valid method for direct ex-vivo quantification of the three main functional subsets of antigen specific T cells. Application to human tuberculosis revealed key immunological differences between distinct clinical stages of MTB infection. The differences between active and latent infection, as well as the novel finding that remote LTBI may be more immunologically distinct from active TB than recent LTBI, delineate functional T cell subsets associated with long-term immune control and protective immunity. T cell functional signatures could potentially serve as biomarkers of risk of progression which, if validated in prospective studies with clinical outcomes [43], [44], could refine targeting of preventive therapy in LTBI. The consistent dynamic changes during treatment, combined with the sensitivity, simplicity and scalability of the robust immunospot platform as exemplified by ELISpot IGRA [45], identify this technique as a promising new tool to monitor TB treatment response in the clinic, evaluate new therapies for TB in clinical trials and assess immunogenicity of new T cell-inducing vaccines in the field [8], [46], [47].

## Supporting Information

Table S1 Camera and count settings used on automated reader for colour-ELISpot and fluorescence-immunospot for all samples studied.
(DOCX)

Table S2 Concordance between assay results from colour-ELISpot and fluorescence-immunospot.
(DOC)
