# Supplementary material for: Enumeration of Functional T-Cell Subsets by Fluorescence-Immunospot Defines Signatures of Pathogen Burden in Tuberculosis
Source: PLoS One. 2010 Dec 14;5(12):e15619. doi: 10.1371/journal.pone.0015619 (PMC3001879; doi:10.1371/journal.pone.0015619)
Supplement: Table S1 — Camera and count settings used on automated reader for colour-ELISpot and fluorescence-immunospot for all samples studied. (DOCX) [file pone.0015619.s001.docx]

|  | IFN-γ colour ELISpot | IL-2 colour ELISpot | Fluorescence-immunospot Optical filter 1 (Cy3) | Fluorescence-immunospot Optical filter 2 (FITC) |
| --- | --- | --- | --- | --- |
| CAMERA SETTINGS |  |  |  |  |
| Brightness | 179 | 179 | 0 | 36 |
| Hue | 141 | 141 | 0 | 51 |
| Saturation | 255 | 255 | 291 | 321 |
| Sharpness | 0 | 0 | 0 | 1 |
| Gamma | 129 | 129 | 0 | 0 |
| White Balance -R | 128 | 128 | 83 | 83 |
| White Balance -B | 125 | 128 | 53 | 53 |
| Gain | 0 | 0 | 32 | 0 |
| Exposure/Shutter | 2286 | 2286 | 1735 | 2746 |
| Autoexposure | 0 | 0 | 125 | 81 |
| COUNT SETTINGS |  |  |  |  |
| Algorithm | V.2.6-2.9 | V.2.6-2.9 | v.3.2.x | v.3.2.x |
| Intensity Minimum | 30 | 28 | 5 | 12 |
| Gradient Minimum | 1 | 1 | 2 | 1 |
| Size Minimum | 40 | 40 | 50 | 50 |
| Emphasis | Big | Big | Tiny | Tiny |

**Table S1**

Camera and count settings used for ELISpot and fluorescence-immunospot.
