# Supplementary material for: Enumeration of Functional T-Cell Subsets by Fluorescence-Immunospot Defines Signatures of Pathogen Burden in Tuberculosis
Source: PLoS One. 2010 Dec 14;5(12):e15619. doi: 10.1371/journal.pone.0015619 (PMC3001879; doi:10.1371/journal.pone.0015619)
Supplement: Table S2 — Concordance between assay results from colour-ELISpot and fluorescence-immunospot. (DOC) [file pone.0015619.s002.doc]

**Table S2**

| **A** |  |  |  | **B** |  |  |
| --- | --- | --- | --- | --- | --- | --- |
| **Number of IFN-γ responses** | | |  | **% of IFN-γ responses** | | |
|  | **Fluorescence-**  **immunospot Positive** | **Fluorescence-**  **immunospot**  **Negative** |  |  | **Fluorescence-**  **immunospot Positive** | **Fluorescence-**  **immunospot**  **Negative** |
| **ELISpot Positive** | 182 | 19 |  | **ELISpot Positive** | 67.4 | 7 |
| **ELISpot Negative** | 11 | 58 |  | **ELISpot Negative** | 4.1 | 21.5 |
|  |  |  |  |  |  |  |
|  |  |  |  |  |  |  |
| **C** |  |  |  | **D** |  |  |
| **Number of IL-2 responses** | | |  | **% of IL-2 responses** | | |
|  | **Fluorescence-**  **immunospot Positive** | **Fluorescence-**  **immunospot**  **Negative** |  |  | **Fluorescence-**  **immunospot Positive** | **Fluorescence-**  **immunospot**  **Negative** |
| **ELISpot Positive** | 152 | 16 |  | **ELISpot Positive** | 56.3 | 5.9 |
| **ELISpot Negative** | 19 | 83 |  | **ELISpot Negative** | 7 | 30.7 |

PBMC from 90 independent samples from 74 donors with treated or untreated TB or with latent TB infection were stimulated with PPD, ESAT-6 or CFP-10 and the number of SFCs was enumerated by single colour IFN-γ and IL-2 ELISpot assays in parallel with the fluorescence-immunospot assay. Responses were scored positively where the number of SFC was more than 2 standard deviations of the mean negative control for each assay above the negative control well for each patient (5 IFN-γ SFCs and 8 IL-2 SFCs more than the negative control well for colour ELISpots and 8 IFN-γ SFCs and 12 IL-2 SFCs more than the negative control well for fluorescence-immunospot). These cut-off points equate to limits of detection of 0.003% of whole PBMC for IFN-γ or dual IFN-γ/IL-2-secreting cells and 0.005% for IL-2-only-secreting cells in the fluorescence-immunospot and are associated with a signal to noise ratio of at least 2:1 where negative control wells contained <9 IFN-γ+ SFCs and <13 IL-2+ SFCs per well (which was the case for 95% of patients).
